# DiabetesSistersVoices: Virtual Patient Community to Identify Research Priorities for Women Living With Diabetes

**DOI:** 10.2196/13312

**Published:** 2019-05-10

**Authors:** Peijin Han, Wanda Nicholson, Anna Norton, Karen Graffeo, Richard Singerman, Steven King, Aditi Sundaresan, Wendy Bennett

**Affiliations:** 1 The Johns Hopkins Bloomberg School of Public Health Baltimore, MD United States; 2 Center for Women’s Health Research, Department of Obstetrics and Gynecology Chapel Hill, NC United States; 3 Public Health Leadership Program, The University of North Carolina School of Medicine and Gillings Global School of Public Health Chapel Hill, NC United States; 4 DiabetesSisters #180, 1112 W Boughton Road Bolingbrook, IL United States; 5 TrustNetMD Washington, DC United States; 6 Johns Hopkins University School of Medicin, Division of General Internal Medicine Baltimore, MD United States

**Keywords:** social media, online social networking, women’s health, diabetes mellitus

## Abstract

**Background:**

Women with or at high risk of diabetes have unique health concerns across their life course. Effective methods are needed to engage women living with diabetes to develop and carry out a patient-centered research agenda.

**Objective:**

This study aimed to (1) describe the creation of DiabetesSistersVoices, a virtual patient community for women living with and at risk for diabetes and (2) assess the feasibility and acceptability of DiabetesSistersVoices for engaging women in talking about their experiences, health care, and research priorities.

**Methods:**

We partnered with a national advocacy organization to create DiabetesSistersVoices and to develop recruitment strategies, which included use of social media, Web-based newsletters, and weblinks through partnering organizations. Study inclusion criteria were as follows: Being a woman aged ≥18 years, residing in the United States, and self-reporting a diagnosis of diabetes or risk of diabetes. Eligible participants were given access to DiabetesSistersVoices and completed online surveys at enrollment and 6 months. We assessed trends in participants’ activities, including posting questions, sharing experiences about living with diabetes, and searching for posted resources.

**Results:**

We enrolled 332 women (white: 86.5%; type 1 diabetes: 76.2%; median age: 51 years [interquartile range: 31 to 59 years]) over 8 months. Most (41.6%, 138/332) were classified as being active users (ie, posting) of the virtual community, 36.1% (120/332) as observers (ie, logged in but no posts), and 22.3% (74/332) as never users (ie, completed baseline surveys but then never logged in). Online activities were constant during the study, although participants had the highest website usage during the first 10 weeks after their enrollment.

**Conclusions:**

We demonstrated the feasibility and acceptability of an online patient community for women living with diabetes by showing durability of recruitment and online usage over 6 months of testing. Next steps are to address barriers to joining a virtual patient community for women of color and women with type 2 diabetes to enhance inclusiveness and gain diverse perspectives to inform diabetes research.

## Introduction

### Background

Diabetes is increasingly common among men and women, affecting 9.4% of the US population [1,2]. Women are disproportionately affected by diabetes, with a greater risk of heart attack and stroke, compared with men with diabetes [3-6]. Compared with women without diabetes, women with diabetes have unique health needs and concerns across their life course, including preconception health, pregnancy, postpartum care, and the menopausal transition. In addition, women with diabetes experience higher rates of eating disorders [6], pregnancy complications [7], sexual dysfunction [8], and higher rates of diabetes-related urinary incontinence [9]. There is a need to understand the patient experience for women living with diabetes to inform and improve patient-centered research, advocacy, and health care delivery for this important, high-risk population.

Increasingly, patients with a variety of conditions, including diabetes, are going online to learn more about their disease, create social support networks, and communicate with other patients such as themselves [10]. In a recent Pew survey, 16% of adult internet users reported going online to find other patients with similar experiences and diagnoses [11]. In fact, there has been a proliferation of online patient communities, including PatientsLikeMe and patient bloggers, especially for people living with and seeking support for living with chronic diseases [12]. The term Web 2.0 was introduced in 2004 to describe the improved communication and collaboration tools available via social networking [13-15]. Growing evidence shows online support groups or “learning health communities” as having benefits for patients [12,16-18]. Along with peer-to-peer support, online patient communities are useful for patient engagement and as an innovative tool to connect patients with researchers so that they can be involved in all stages of the research process [19,20].

### Study Objectives

Engaging patients about what is most important to them is crucial to direct researchers, consumer and advocacy organizations, policy makers, health systems leaders, and funders toward clinical research and health system improvements that are patient-centered and meaningful [21]. Our goal was to develop a virtual patient community for women with diabetes to communicate with each other about their experiences to help identify research and health care priorities.

In partnership with DiabetesSisters, a national advocacy organization for women living with any type of diabetes, we developed and launched DiabetesSistersVoices, a virtual patient community to engage women with diabetes to talk about patient-centered research priorities and create a platform for peer support. We aimed to engage women to join the community across racial/ethnic groups, geographical regions, and types of diabetes. The objectives of this study were to (1) describe the creation of DiabetesSistersVoices, a virtual patient community for women living with and at risk for diabetes and (2) assess the feasibility and acceptability of DiabetesSistersVoices for engaging women about their experiences, health care, and research priorities.

## Methods

We began with the development phase and refinement of the website, followed by a recruitment and enrollment phase to engage women with diabetes. The institutional review boards at Johns Hopkins University and the University of North Carolina approved the study.

### Phase 1: Development and Refinement of DiabetesSistersVoices

The DiabetesSistersVoices virtual patient community’s platform began with refining a recently developed and tested platform. The platform, developed by Lehmann and colleagues [22], had been built for communication between community health workers. We chose this platform’s software approach because it utilized open-source software with customized interfaces and modules, making it easily adaptable and scalable, and it already contained multiple features that enabled communication and information sharing. Several key features of the platform were the ability to post a question or topic, respond to other posts, search for or post resources for others, tag or relabel, or “like” the topics. “Liking” a post provided points for the post to show its popularity or helpfulness.

As an initial step to refining the platform for the needs of women with diabetes, the chief executive officer of DiabetesSisters (AN) invited 8 patients and advocates who were active in the organization and willing to donate time to participate in semistructured phone interviews with the platform developer and research team. We demonstrated the functions of the platform and inquired about their desired functionalities and features for the new site.

We convened a diverse stakeholder advisory board to gather early input about the design and launch of DiabetesSistersVoices [23]. Stakeholder advisory board members represented 5 stakeholder groups including patient partners from DiabetesSisters, a national nonprofit diabetes organization for women and our main partner in this study. We additionally included leaders from the Black Women’s Health Imperative and PatientsLikeMe and a researcher conducting community-based work related to diabetes in racial minority communities. Members met every 3 months and they provided insights and feedback about the platform’s clarity and usability, font size, white space, and images and instructions for participants during the meetings. To ensure broad applicability of the platform, we iteratively refined the Web pages by conducting one-on-one “hands-on” semistructured interviews with each stakeholder advisory board member and additional patient partners identified by the stakeholder advisory board’s representative organizations (Multimedia Appendix 1 contains the interview guide). Comments from the semistructured interviews were presented back to the research team and used to refine the Web platform. From these interviews, we gathered valuable feedback on the ease of use of traversing the website and the ability to express ideas and exchange information with other participants.

**Figure 1:**
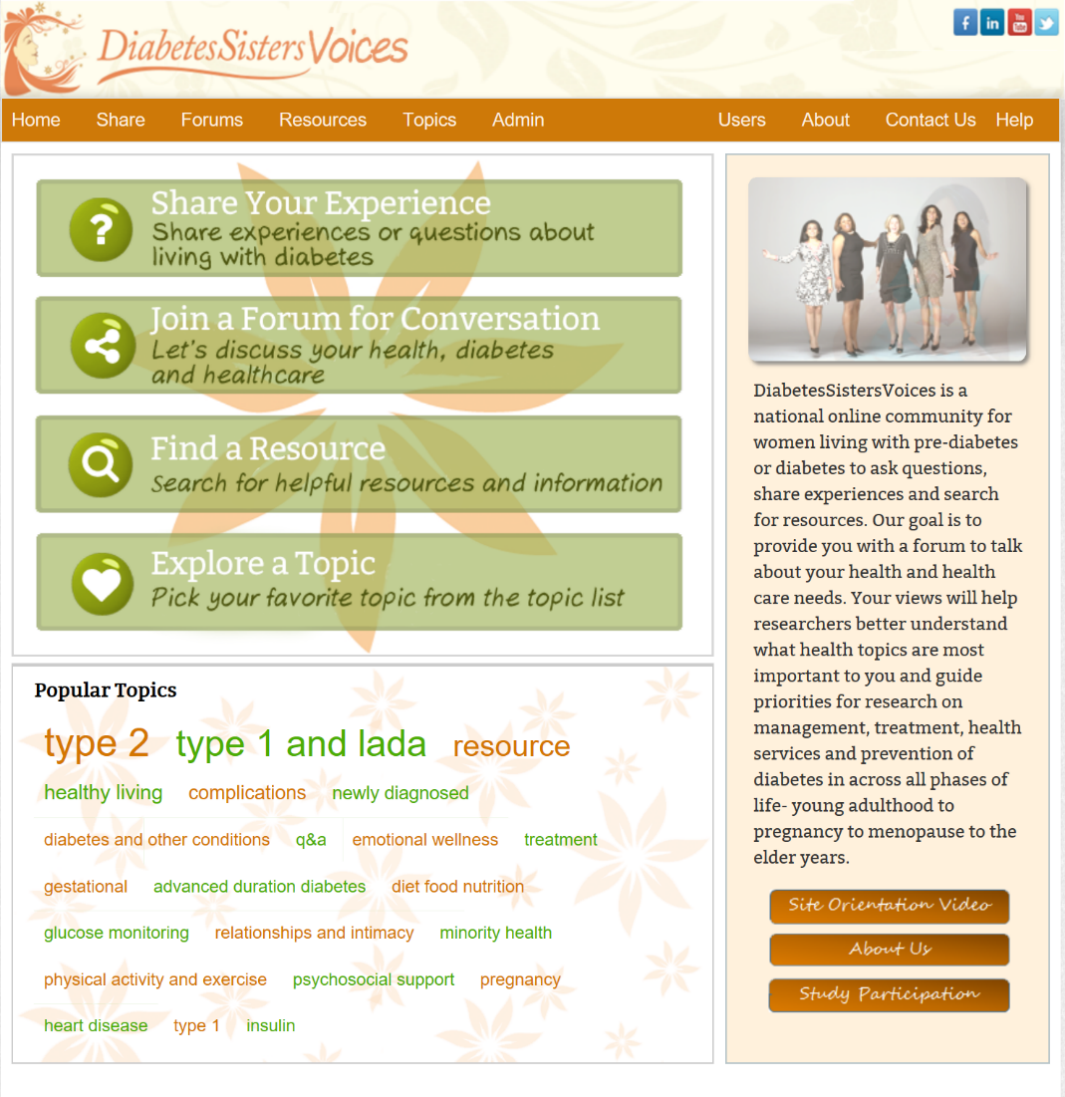
Landing page of DiabetesSistersVoices. There were 4 basic functions of the website, including “share your experience,” “join a forum for conversation,” “find a resource,” and “explore a topic”. The size of popular topics listed below was based on their popularity, that is, the number of times they were clicked.

The final version of the website used in Phase 2 (www.diabetessistersvoices.org) comprised multiple features to facilitate online conversation. Additional features beyond those in the original platform included (1) a video orientation guide for participants that reviewed the goals of the project and how to use the site and (2) an online moderator who monitored discussions (and could take down posts if not appropriate), welcomed participants, and sent weekly emails (see Multimedia Appendix 2 for the list of topics). Figure 1 shows the landing page of DiabetesSistersVoices that participants were able to view after completion of the sign-in process using their individually selected username and password.

### Phase 2: Observational Cohort Study

In Phase 2, we recruited women with diabetes to use the DiabetesSistersVoices virtual patient community over 6 months.

### Recruitment Strategies

In collaboration with the stakeholder advisory board, we developed and employed a wide range of recruitment strategies and activities to attract women to the site and engage participants across different age, racial, and socioeconomic strata. The recruitment strategies included (Multimedia Appendix 3):

1. Social media promotion through Facebook and Twitter and monthly electronic newsletters from DiabetesSisters. Facebook posts were “boosted” monthly to showcase the posts to more users.
2. Printed brochures, posters, and postcards distributed in person at churches in Baltimore, health clinics, health fairs, and conferences for patients with diabetes.
3. A toolkit aimed at partnering organizations, which included a template for the invitation from the partner, a description of DiabetesSistersVoices, and promotional materials including flyers and postcards.
4. The site moderator used online “Q&A” threads to keep promoting new discussion topics and emailed a weekly topic to all the registered users to get them to come back (see Multimedia Appendix 2 for the list of weekly topics).

### Study Participants: Eligibility and Online Consent Process

Participants registered to participate on the DiabetesSistersVoices website. The website contained information about the project; and if they were interested, they clicked “Register to Participate” and were prompted to provide their name and email address. Our study team received a daily list of emails from the website. Email addresses were entered into the QUALTRICS survey platform to send an email message with further details about the study and a unique link to online screening questions. Participants were unable to access this unique link again, once consent was completed.

Study inclusion criteria were of the female gender, aged ≥18 years, and self-report of type 1, 2, or prediabetes (defined as “at risk for diabetes” or gestational diabetes mellitus history) and residing in the United States. Following successful completion of the screening questions, participants completed an electronic consent process and downloaded a copy of the consent form. We conducted a monthly raffle of items related to healthy living with diabetes (eg, hand weights, yoga mats, and books) to participants who logged in ≥2 times in the past month, starting December 2016.

### Data Collection

We assessed with 2 time points of data collection using online surveys (at baseline enrollment and after 6 months since the launch of the website) and had continuous surveillance of website utilization using Google Analytics, a Web analytics service.

### Online Surveys

Participants completed a baseline survey following consent. Participants who had been enrolled for ≥1 month completed an end of study online survey (administrated 6 months after the initiation of the study). Participants received surveys as an email with a unique survey link. Participants were able to review and change their answers through a back button and then click submit, and the survey completeness was further verified after it was submitted. Survey response data were captured and stored automatically using the Johns Hopkins QUALTRICS server, which was protected under the Johns Hopkins University firewall, and only the principal investigators, coinvestigator, and study coordinator had access to the data. Baseline survey questionnaires assessed sociodemographic characteristics, health status, internet use, social support, and health-related quality of life using standard measures (Multimedia Appendix 4) [24,25]. We used the Medical Outcomes Study Social Support Survey Instrument to assess emotional and informational social support [26] and the Patient-Reported Outcomes Measurement Information System (PROMIS) scale version 1.2 Global Health survey instrument to assess users’ self-reported health status [27]. The end of study surveys contained the Medical Outcomes Study (MOS) Social Support Survey instrument, PROMIS scale version 1.2 Global Health survey instrument, and satisfaction with the site (see Multimedia Appendix 5 for site satisfaction).

To measure the utilization of the DiabetesSistersVoices online community, we used Google Analytics, a Web analytics service, to track and report DiabetesSistersVoices’ website traffic and activities, including the length of time of individual sessions, number of downloads, page views, “clicks” on topic tags, and “likes” that were captured by this application. We were able to link website users with survey data using a deidentified unique user identification number, assigned by Google Analytics at the time of registration.

### Data Analysis

We defined “never users” as participants who enrolled for the study but never logged in to the community, “observers” as participants who logged in to the site at least once but never posted on the site, and “active users” as participants who posted on the site at least once. We reported engagement activities as “posting” comments, “liking” comments or resources, clicking on a topic, and conducting a search. We calculated the proportion of participants who engaged in each of these activities at least once over the previous 2 weeks.

We used descriptive statistics to describe baseline characteristics of participants over time and by level of online engagement. We presented descriptive statistics for the satisfaction level of the participants who completed the end of study satisfaction survey. For participants who were eligible to complete the online survey (ie, enrolled in the study for at least 1 month), we compared the sociodemographic of those who completed the end of study social support and health-related quality of life survey with those who did not. Both completed questionnaires and questionnaires terminated early were included in the analysis.

To test for statistically significant differences between groups, we performed a nonparametric k-sample test on the equality of medians for continuous variables and chi-square test for categorical variables. A 2-sided *P* value ≤.05 was considered statistically significant. All the statistical analyses were performed using STATA (StataCorp LLC).

## Results

### Enrollment into DiabetesSistersVoices Virtual Patient Community

**Figure 2:**
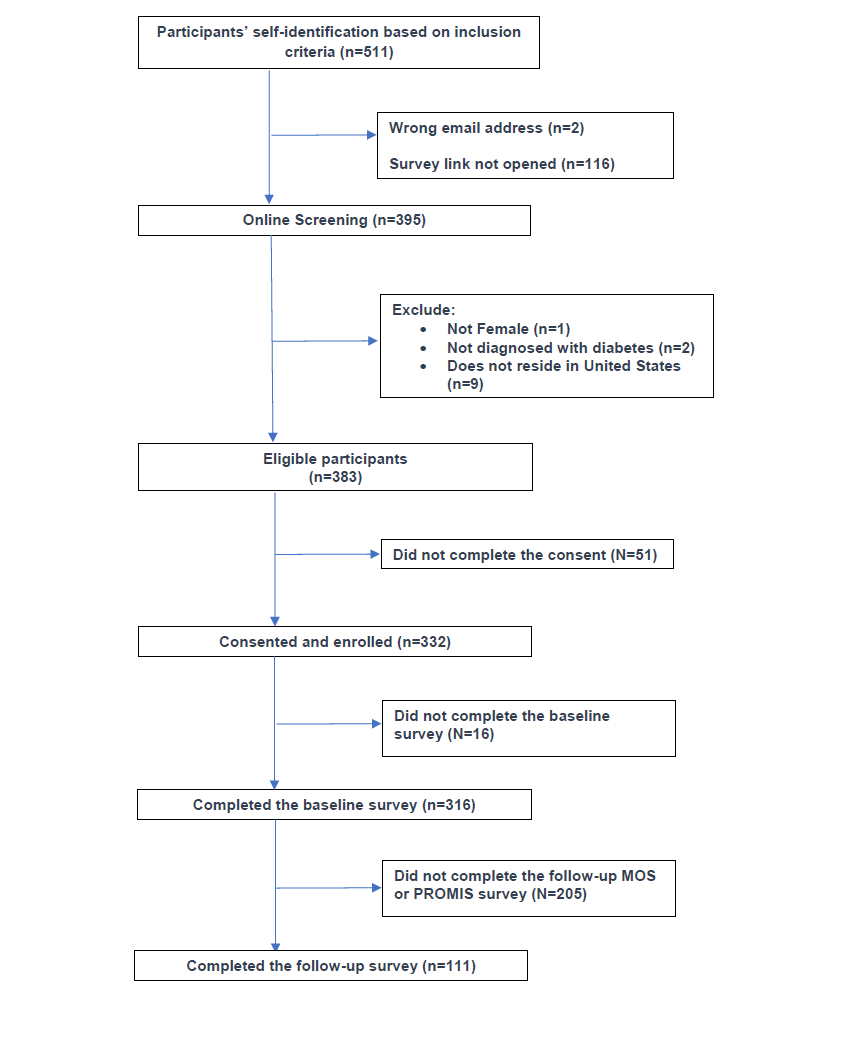
Enrollment flow of participants into the DiabetesSistersVoices study. MOS: Medical Outcomes Study; PROMIS: Patient-Reported Outcome Measurement Information System.

From November 2016 to June 2017 (30 weeks), 511 women registered at the DiabetesSistersVoices website. Among them, 395 women began the online screening and consent process and 332 women completed the consent form and enrolled in the study (Figure 2).

**Figure 3:**
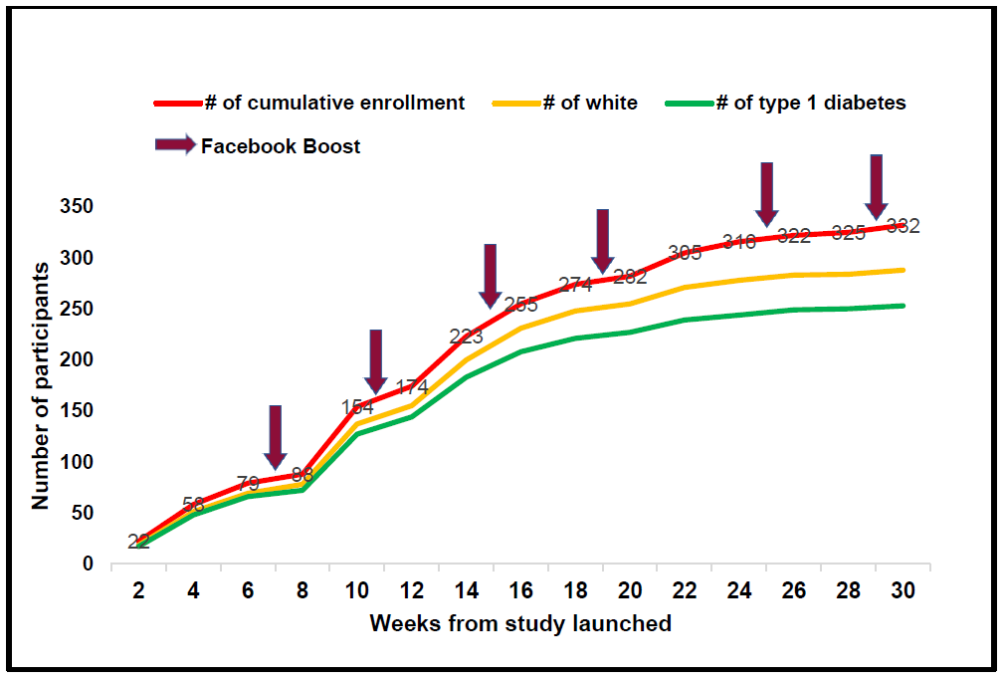
Participants’ enrollment over time–overall, by race, and type of diabetes. Red arrows indicate the time of Facebook boost.

Figure 3 demonstrates the cumulative enrollment of participants over the 30 weeks, overall, and stratified by race and diabetes type (type 1 vs type 2 diabetes). At the beginning of the study, all participants were white with type 1 diabetes, when the majority of promotions were by DiabetesSisters’ online and in-person (at conferences) advertising. Over time and with the expansion of recruitment methods, the diversity of study participants increased. The study was “boosted” on Facebook multiple times for women with interests in diabetes-related topics.

### Participant Characteristics

Table 1 shows the baseline characteristics of study participants who enrolled in the DiabetesSistersVoices virtual patient community. Overall, participants were 86.5% (287/332) white, 6.3% (21/332) black, 3.3% (11/332) Hispanic, 0.9% (3/332) Asian, and 3.0% (10/332) elected not to report race. The median age was 51 years (interquartile range [IQR]: 37 to 59). Compared with the “never users” and “observers,” “active users” were older (aged 54 years vs 49 and 50 years; *P*=.04). A total of 68.4% (225/332) of the participants had a college degree or above, followed by 30.4% (100/332) who had high school or some college, and 1.2% (4/332) had less than a high school diploma. The majority (76.2%, 253/332) of participants had type 1 diabetes (vs 22.0% [73/332] with type 2 diabetes). No participants reported having “prediabetes.” On average, participants were in the study for 5 months (IQR: 4 to 6 months). Most women reported frequent email use of at least once daily (90.0%, 297/332) and use of social networks such as Facebook (89.9%, 295/332).

A total of 74 (22.3%) of participants were classified as “never users,” 120 (36.1%) as “observers,” and 138 (41.6%) as “active users.” Similar proportions of white and nonwhite women and women with type 1 (vs type 2 diabetes) were active users on the site (Table 1). Compared with “never users,” “observers” and “active users” were more likely to use email daily (93.3% and 91.3% vs 82.2%) and use social networking sites (93.2% and 90.0% vs 84.7%). “Active users” had higher baseline social support scores compared with “never users” and “observers,” but there was no difference in the quality of life scores (Table 1).

**Table 1:** Characteristics of study participants who enrolled in the DiabetesSistersVoices virtual patient community.

Participants’ characteristics		All users (n=332)	Never users (n=74)	Observers (n=120)	Active users (n=138)	*P* value^a^
Age (years), median (interquartile range)		51 (37-59)	49 (34-56)	50 (36-58)	54 (40-63)	.04
**Education^b^** **, n (%)**						
	Less than high school or GED	4 (1.2)	2 (2.7)	0 (0.0)	2 (1.5)	.13
	High school or some college	100 (30.4)	29 (39.2)	31 (26.1)	40 (29.4)	.13
	College degree and above	225 (68.4)	43 (58.1)	88 (74.0)	94 (69.1)	.13
**Race/ethnicity^b^** **, n (%)**						
	White	287 (86.5)	61 (82.4)	104 (86.7)	122 (88.4)	.27
	Black	21 (6.3)	8 (10.8)	7 (5.8)	6 (4.4)	.27
	Asian	3 (0.9)	2 (2.7)	0 (0.0)	1 (0.7)	.27
	Hispanic	11 (3.3)	2 (2.7)	5 (4.1)	4 (2.9)	.27
	Elected not to report	10 (3.0)	1 (1.4)	4 (3.3)	5 (3.6)	.27
**Diabetes type^b^** **, n (%)**						
	Type 1	253 (76.2)	53 (71.6)	90 (75.0)	110 (79.7)	.45
	Type 2	73 (22.0)	20 (27.0)	26 (21.7)	27 (19.6)	.45
Diabetes treatment duration (years), mean (SD)		22.0 (15.0)	21.7 (14.6)	20.5 (13.2)	23.6 (16.1)	.18
**Email use^b^** **, n (%)**						
	Daily	297 (90.0)	60 (82.2)	111 (93.3)	126 (91.3%)	.08
	Every few days	30 (9.1)	11 (15.1)	8 (6.7)	11 (8.0%)	.08
	Less than weekly	3 (0.9)	2 (2.7)	0 (0.0)	1 (0.7%)	.08
Ever use social networking sites^b^, n (%)		295 (89.9%)	61 (84.7)	110 (93.2)	124 (90.0)	.17
**Website engagement**						
	Number of sessions, median (IQR)	3 (1.0-5.5)	—^c^	2 (1.0-4.0)	5.5 (4.0-12.0)	<.001
	Total topic clicks	421	—	96	325	—
	Total posts/responses	904	—	0	904	—
	Total likes	530	—	42	488	—
	Total searches	167	—	32	135	—
	Total downloads	671	—	121	550	—
Total social support score, mean (SD)^d^		68.0 (23.6)	64.3 (25.6)	71.1 (22.5)	67.2 (23.8)	.22
PROMIS-physical health score, mean (SD)^d^		36.4 (4.7)	36.7 (4.5)	36.1 (4.0)	36.4 (5.2)	.68
PROMIS-mental health score, mean (SD)^d^		38.4 (8.7)	39.4 (7.8)	38.0 (8.6)	38.4 (9.1)	.28

^a^Nonparametric k-sample test on the equality of medians for continuous variables and chi-square test for categorical variables.

^b^Percentage may not add up to 100% because of missing data.

^c^—Not applicable.

^d^A total of 316 out of 332 completed the Patient-Reported Outcomes Measurement Information System (PROMIS) Quality of Life and Medical Outcomes Study (MOS) social support questionnaires. MOS scores range from 0 (lowest) to 100 (highest). PROMIS scores were presented as *t* score. The norm in population is mean 50 (SD 10). Scores 0.5 to 1.0 SD or worse than the mean=mild symptoms/impairment, scores 1.0 to 2.0 SD or worse than the mean=moderate symptoms/impairment, and scores 2.0 SD or worse than the mean indicate more severe symptoms/impairment.

### Online Activities on DiabetesSistersVoices Virtual Patient Community

**Figure 4:**
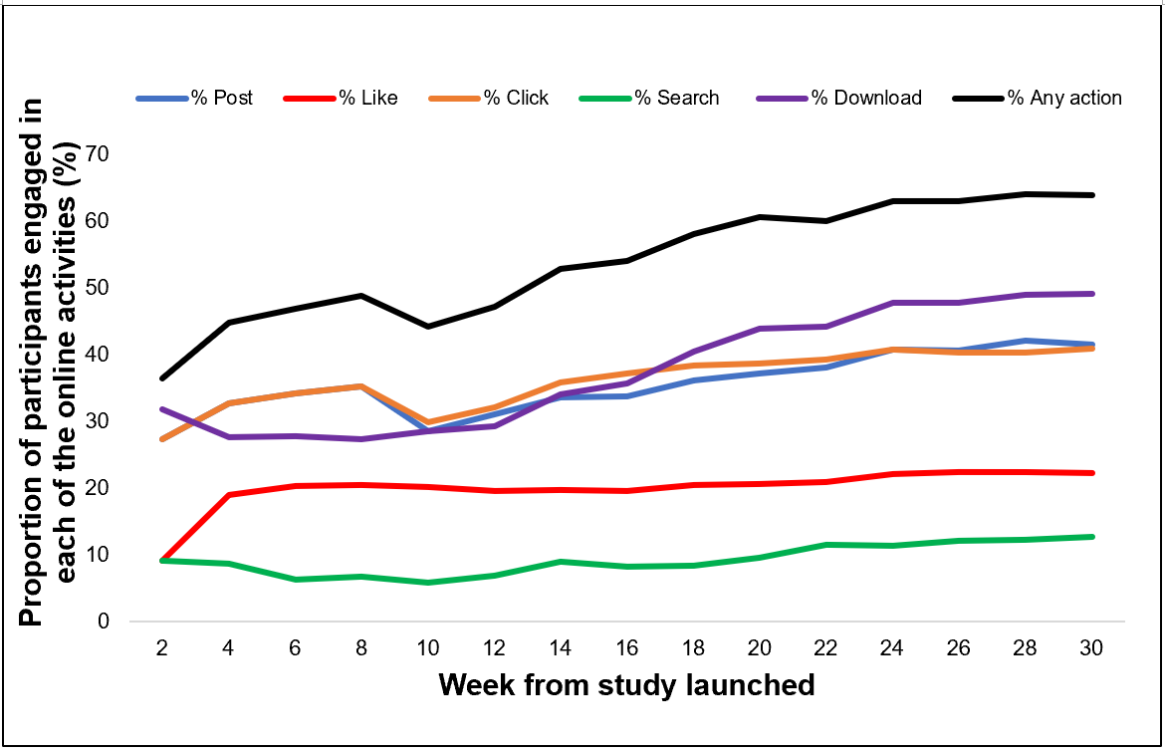
Participants’ online activities over time. The proportion of participants who were engaged in each of the online activities was accumulatively calculated every 2 weeks since the initiation of the study.

**Figure 5:**
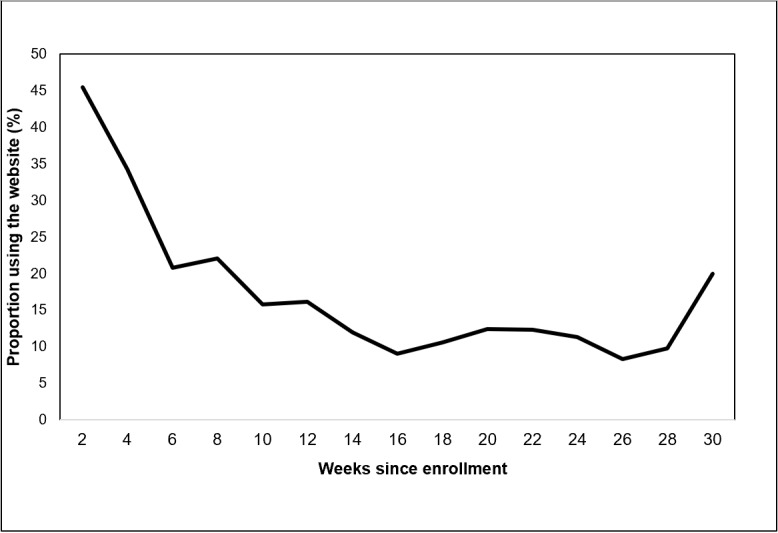
Proportion of participants engaged over time (in weeks) following their study enrollment. The numbers below the x-axis represent the total number of participants in the study.

Over the 30-week study, study participants clicked on topics 421 times, posted or replied on the site 904 times, “liked” posts 530 times, searched for resources 167 times, and downloaded resources 671 times (Table 1). Figure 4 shows that online activities on the site were constant during the study, with about one-third of participants at any given time posting or clicking a topic on the site at least once every other week. Fewer participants “liked” posts on the site (approximately 20%) or performed searches (<10%). Figure 5 shows the proportion of participants engaged over time following their enrollment into the study. On average, participants’ website usage was highest during their first 10 weeks of enrollment. A total of 10 participants were enrolled during the entire study duration of 30 weeks.

### Satisfaction With DiabetesSistersVoices Virtual Patient Community

Table 2 describes the responses for the end of study satisfaction survey, completed by 120 out of 323 eligible participants (37.2%). A total of 67.5% of these participants met the definition of “active users” (Table 2). The majority (94%) of participants were not satisfied with the electronic consent process. However, participants were generally satisfied with the major functions of the DiabetesSistersVoices platform, including logging in, posting questions, searching for comments, and learning topics from weekly email sent by the site moderator. Active website users tend to have a higher satisfaction level compared with the “never users” (data not shown).

Compared with participants who completed only the baseline social support and quality of life survey, participants who completed both baseline and end-of-study surveys were senior (aged 54 years vs 49 years; *P*<.001) and had a higher education level (Multimedia Appendix 6). The distributions of race and diabetes diagnosis had no statistically significant difference between those 2 groups (Multimedia Appendix 6).

**Table 2:** Participants’ satisfaction with the study and DiabetesSistersVoices virtual patient community’s platform features (N=120).

Aspects of the DiabetesSistersVoices virtual patient community		Statistics, n (%)
**Consenting to participate in the study^a^**		
	Dissatisfied	21 (17.5)
	Neutral	92 (76.7)
	Satisfied	7 (5.8)
**Online survey about you and your health at the start of the study**		
	Dissatisfied	1 (0.8)
	Neutral	29 (24.2)
	Satisfied	80 (66.7)
**Logging on to the website**		
	Dissatisfied	16 (13.3)
	Neutral	17 (14.1)
	Satisfied	82 (68.3)
**Posting questions or comments**		
	Dissatisfied	16 (13.3)
	Neutral	24 (20.0)
	Satisfied	66 (55.0)
**Searching for resources**		
	Dissatisfied	14 (11.7)
	Neutral	22 (18.3)
	Satisfied	67 (55.8)
**Communicating with other members of the community**		
	Dissatisfied	15 (12.5)
	Neutral	25 (20.8)
	Satisfied	62 (51.7)
**Topic of weekly email**		
	Dissatisfied	11 (9.2)
	Neutral	24 (20.0)
	Satisfied	76 (63.3)

^a^Percentage may not add up to 100% because some participants did not use the feature.

## Discussion

### Principal Findings

In partnership with DiabetesSisters, a national organization serving women living with diabetes, and a diverse stakeholder advisory board, we created the DiabetesSistersVoices virtual patient community and enrolled 332 women with all types of diabetes to provide peer support and to identify high priority research areas for women living with diabetes. Our results demonstrate strong feasibility and acceptability of the online patient community, showing high levels of website engagement, even over a short time period. However, like other “online” only studies, the major limitation was the lack of broad representation of women living with diabetes (ie, older women, women of color, and women with type 2 diabetes). In addition, participants reported high levels of satisfaction with the DiabetesSistersVoices virtual patient community but were less satisfied with the electronic consent for research, which may have created a barrier for those who never enrolled.

### Advantages of and Barriers to Online Patient Engagement

Virtual patient communities have multiple purposes, including peer-to-peer patient support (eg, PatientsLikeMe) [12,20,28], dissemination and sharing of resources either coming from health professionals or from patients to other patients, [12,29,30], as well as recruitment into research studies [31]. In addition, like DiabetesSistersVoices, virtual patient communities can provide an opportunity for researchers to engage with patients about what issues are most important to them for ongoing or to guide future research [12,19,32,33]. Patients value peer-to-peer communication as being “more real.” In a Web-based smoking cessation trial, the “crowdsourced” messages by patients about how to quit smoking were associated with greater return visits to the smoking cessation clinic [34]. However, most patient engagement websites are not moderated, and we identified that patient-led moderation is an important component of patient engagement. To encourage engagement and re-engagement, the site moderator used online “Q&A” threads to keep promoting new discussion topics and emailed a weekly topic to all the registered users to encourage them to come back (Multimedia Appendix 2 lists the weekly topics). In addition, growing evidence supports virtual support groups or “learning health communities” as having some benefits for patients [12,16,17,35,36]. A 2004 systematic review identified 38 studies reporting computer-based peer-to-peer communities, and electronic self-support groups showed a need for rigorously designed studies to evaluate the effects of social media interventions on health behaviors and long-term health outcomes [36]. Given the strength of evidence supporting *in-person* peer-to-peer support for complex health behaviors and disease management in a wide variety of settings and disease [37,38]. In addition, few studies have used electronic modalities for patient engagement around research and to identify research priorities [21].

Despite our goal to engage diverse women with both type 1 and type 2 diabetes, the majority of DiabetesSistersVoices participants were of white race and had type 1 diabetes, likely because of our strong affiliation with the DiabetesSisters organization, which has a strong allegiance and trust within the community of women with type 1 diabetes. Given the disparities in the diabetes epidemic [39], there is a high need to develop Web-based engagement strategies specifically tailored for African American and Latina women [40,41] as well as women with type 2 diabetes and prediabetes [42]. African American women with diabetes are diverse with regard to age, region (urban and rural population), access to care, socioeconomic status, and types of health disparities they experience. For example, diabetes self-management interventions have been designed and specifically tailored for African American women to take into account women’s values, multiple caregiving roles, cultural implications around eating and weight, socioeconomic barriers to healthful eating and medication adherence, and spiritual and social support needs, as well as day-to-day barriers to self-management (eg, high stress, family conflicts, and coping styles) [43-45]. Web-based modalities also need to specifically address the needs and values of African American women to have greatest impact.

Online engagement of African American women using Web-based modalities is underutilized in diabetes research despite the fact that African American women are “connected” and use mobile technology at very high rates [46]. In 2014, African Americans trailed whites by only 7% in internet use (80% vs 87%, respectively) [47] and the gap continues to close [48]. However, among them, compared with the traditional recruiting strategies (eg, paper flyers, media, and face-to-face advertising), the online enrollment rate was proportionally smaller [49]. Other studies have identified barriers to online recruitment for African Americans to enroll into research studies, include a learning curve for computer usability [50], preference to face-to-face support [51], and mistrust of the researchers [51-53]. In addition, barriers specific to diabetes, such as “diabetes stigma” ie, diabetes viewed as a “lifestyle disease” with blaming of the individual), may also prevent women to self-identify (eg, discuss their diagnosis online), seek out support and resources, and join a virtual peer community with other patients [54-58].

### Study Limitations

There are several limitations to this study. First, because this was a research study, the consent process posed a barrier to women who otherwise may have joined an online peer support group. Second, we had lower enrollment in the study for minority women and women with type 2 diabetes. As described above, the majority of our participants were white and with type 1 diabetes, in part because our main stakeholder partner, DiabetesSisters, has a community in which 65% are women living with type 1 diabetes. We also engaged fewer older women who may use the internet for information but not as a method for social interaction with peers. Third, this was a short-term study, totaling 6 months, but many women used the site for less than 6 months (range 1 to 6 months), limiting our ability to assess changes in social support or diabetes knowledge as a results of website participation. Finally, overall rates of “posting” new content on the site was low but consistent with typical Web behaviors described on other websites, where the majority of users are considered “observers” but not active posters or leaders in social networking sites [59-61].

### Conclusions

In summary, our study findings suggest that a virtual patient community can be an engaging and efficient tool for women with diabetes to interact with each other and provide their perspectives about diabetes care to inform the next generation of research questions. We identified multiple approaches to engage women to share their perspectives on a range of topics and interest related to diabetes. Further study is needed in a larger cohort of women with type 2 and gestational diabetes as the majority of study participants had type 2 diabetes. Furthermore, future studies might consider targeting aspects of diabetes care (eg, fertility and menopause) that are specific to women and address conditions across the woman’s lifespan.

## Appendix

Multimedia Appendix 1 Instructions for DiabetesSistersVoices users: interview guide.

Multimedia Appendix 2 Topic list in April newsletter.

Multimedia Appendix 3 Examples of social media and recruitment materials. Exhibit from left to right: a Facebook post, a flyer used online and for in-person recruitment, and the title page for the Toolkit we distributed to stakeholders with information about the study and recruitment materials.

Multimedia Appendix 4 DiabetesSistersVoices baseline survey questionnaires.

Multimedia Appendix 5 DiabetesSistersVoices end of study satisfaction survey.

Multimedia Appendix 6 Comparison of DiabetesSistersVoices study participants’ characteristics--those who only completed baseline social support and quality of life survey (and not end of study survey), N=205, compared with participants who completed both baseline and end of study social support and quality of life survey N=111.

## Abbreviations

IQR: interquartile range
MOS: Medical Outcomes Study
PROMIS: Patient-Reported Outcomes Measurement Information System
