# Supplementary material for: DiabetesSistersVoices: Virtual Patient Community to Identify Research Priorities for Women Living With Diabetes
Source: J Med Internet Res. 2019 May 10;21(5):e13312. doi: 10.2196/13312 (PMC6533875; doi:10.2196/13312)
Supplement: Multimedia Appendix 1 [file jmir_v21i5e13312_app1.docx]

On behalf of your DiabetesSistersVoices team, thank you for partnering with us as we enter into this exciting phase of this project to test the DiabetesSistersVoices website for future users. In this phase of the project, we ask that you participate online as users so we can get your insights and opinions. Your expertise and experiences are very important to this project.

# What is the goal of this website?

To create an interactive, engaging online community for women with diabetes to provide input about what is important for research and healthcare to improve their health and well-being.

# How do I register?

- Click this link http://diabetes-sisters-voices.org/home to go to the DSVoices test site and “Register” by clicking on that word in the top banner and completing the form. Upon completing the registration form, you will have access to the site.

# What should I do online?

- **PLAY**: Over the next 3 to 4 days, please go to the site at least once a day and actively participate and engage in mock sessions on the site by doing the 4 main functions (ASK, SHARE, FIND, & JOIN) which are displayed on the HomePage as well as tabs at the top. Take notes on what you like and dislike.
- **ACCESS SITE DIFFERENTLY:** Use various devices to access the site (e.g. desktops/laptops, tablets, & mobile devices). Take notes on any issues which appear to be due to the type of device used.
- Please ask if anything is not clear

# Please provide is with feedback about how to improve the site

- Participate in a **30-45-minute interview** with one of our team members where you will share your notes and where you will be asked about 1) how easy or hard it is to use the different functions of the site; 2) the resource materials that are included on the site and 3) the ease in following the instructions to use the site.

**To help guide you while you are on the site (*feel free to use this outline for taking notes*), here is the type of feedback we are looking for you to provide during the interview:**

1. FEEDBACK QUESTIONS:
   1. **Overall impression:**
      1. Enticing – friendly?
      2. Interactive, engaging?
      3. Usability, easy to maneuver around the site?
      4. Were their issues using different devices?
   2. **Layout & Design:**
      1. How easy is it to follow the layout of the DiabetesSistersVoices site?
      2. Font size – easy to read?
      3. Formatting – enhanced the reading?
      4. Color scheme – pleasing to the eye?
      5. Clarity of images & text – self-explanatory to guide you what to do?
   3. **Input & User Comments:**
      1. Was inputting an item simple & self-explanatory?
      2. “Ask” function was clear as to what it was?
      3. “Share” function was clear as to what it was?
      4. How easy was it to find a topic (issue) you wanted to comment on?
      5. Was it easy or hard to understand comments of other women?
      6. Was it easy or hard to rank a topic as priority?
      7. Were you motivated to go back to the site when you received the email notifications on comments?
   4. **Resources:**
      1. Do the **resources** provide useful information?
      2. Easy to find what you were looking for?
      3. What resources might be missing?
   5. **Searching:**
      1. Tell us about your experience in searching?
         1. Find function
         2. Search bar
         3. Word Cloud
         4. Topic page
   6. What did you **like most** on the site?
   7. What did you **like least** on the site?
