# Supplementary material for: DiabetesSistersVoices: Virtual Patient Community to Identify Research Priorities for Women Living With Diabetes
Source: J Med Internet Res. 2019 May 10;21(5):e13312. doi: 10.2196/13312 (PMC6533875; doi:10.2196/13312)
Supplement: Multimedia Appendix 2 [file jmir_v21i5e13312_app2.docx]

## **Supplement B: Examples of Weekly Email Topics**

##

## Here is what’s happening at DiabetesSistersVoices this week. Come join the conversation!

First Drop or Second? - When checking your blood sugar, do you use the first drop of blood, or wipe it away and use the second drop? Join our discussion here.

Exercise and Weight Loss with an Insulin Pump – Share your reliable resources and advice on pump adjustments to avoid lows while exercising.

Come see the Resource of the Week:

Type 2 Diabetes Oral Medications – Check out this comprehensive guide from the DiabeticLifestyle site.

DON’T MISS THIS:

The Birth Control Pill and Type 1 Diabetes – Is the pill safe for women with diabetes, and does it affect blood sugars? Tell us your experience here.
