# Supplementary figures and images for: DiabetesSistersVoices: Virtual Patient Community to Identify Research Priorities for Women Living With Diabetes
Source: J Med Internet Res. 2019 May 10;21(5):e13312. doi: 10.2196/13312 (PMC6533875; doi:10.2196/13312)

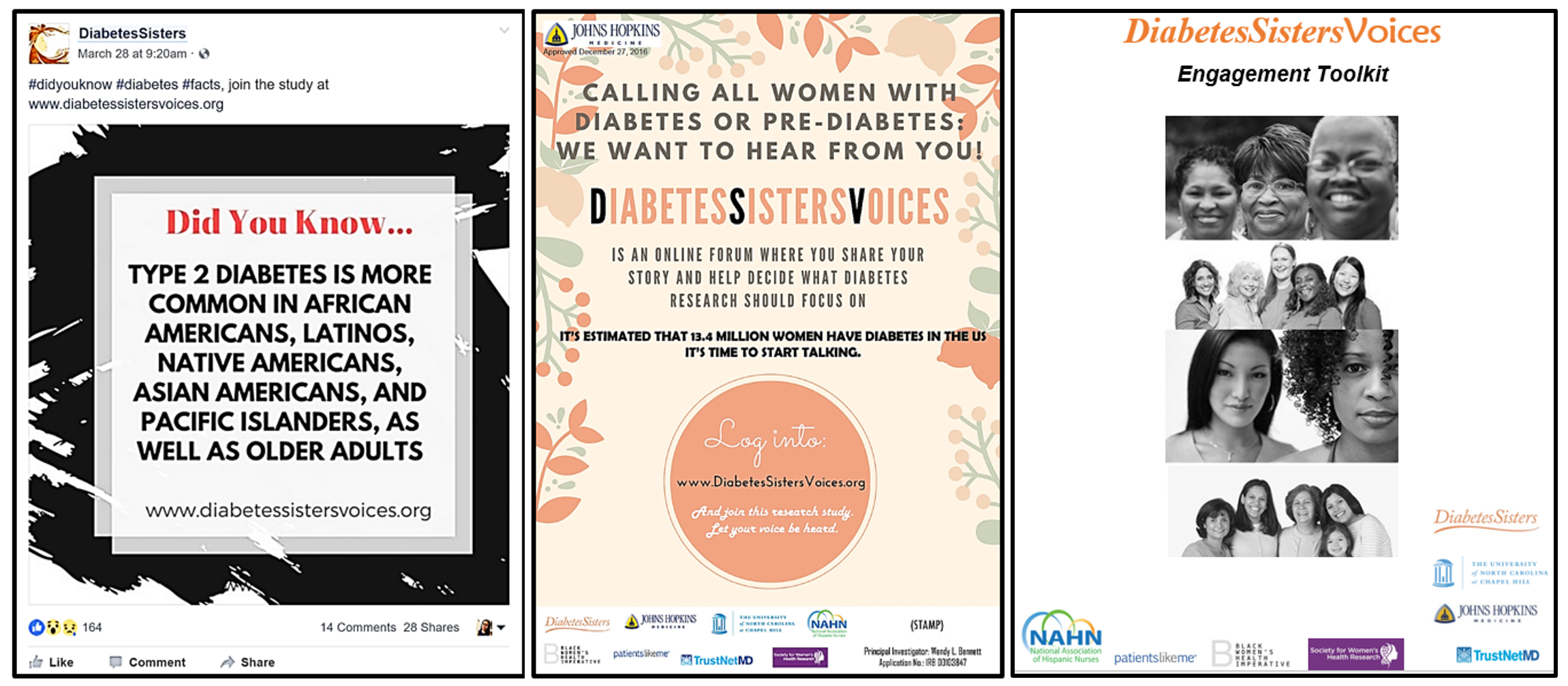

Supplement: Multimedia Appendix 3 [file jmir_v21i5e13312_app3.PNG]
