# Supplementary material for: DiabetesSistersVoices: Virtual Patient Community to Identify Research Priorities for Women Living With Diabetes
Source: J Med Internet Res. 2019 May 10;21(5):e13312. doi: 10.2196/13312 (PMC6533875; doi:10.2196/13312)
Supplement: Multimedia Appendix 4 [file jmir_v21i5e13312_app4.docx]

**Supplement D.** DiabetesSistersVoices Baseline survey questionnaires

**Section A: About you (page 1):**

1. What State do you live in? (drop down)
2. What is your zip code? __ __ __ __ __
3. What is the highest degree or level of school you have completed?
4. 8th grade or less
5. Some high school, but did not graduate
6. High school graduate or GED
7. Some college or 2-year degree
8. College graduate
9. More than a college degree
10. Prefer not to answer
11. What is your race or ethnicity? (check all that apply)
    1. Asian (i.e. Asian Indian, Chinese, Filipino, Japanese, Korean, Vietnamese, Hmong, Laotian, Thai, Pakistani, Cambodian, etc.)
    2. Black, African American, African, or Afro-Caribbean (i.e. African American, Haitian, Nigerian, etc.)
    3. Hispanic, Latino, or Spanish origin (i.e. Mexican, Mexican American, Puerto Rican, Cuban, Argentinian, Colombian, Dominican, Nicaraguan, Salvadorian, Spaniard, etc.)
    4. Middle Eastern/North African
    5. Native American, American Indian or Alaskan Native (i.e. Navajo, Mayan, Tingt, etc.)
    6. Native Hawaiian or Other Pacific Islander (i.e. Native Hawaiian, Guamanian or Chamorro, Samoan, Fijian, Tongan, etc.)
    7. White (i.e. German, Irish, Lebanese, Egyptian etc.)
    8. Some other race or origin *(please specify)____________________________*
    9. Prefer not to answer
12. What language do you mainly speak at home?
    1. English
    2. Spanish
    3. Chinese
    4. Another other language (please print):___________________________
    5. Prefer not to answer
13. Are you currently (check all that apply):
    1. Employed for wages (full-time or part-time)
    2. Self-employed
    3. Out of work for more than 1 year
    4. Out of work for less than 1 year
    5. A homemaker
    6. A student
    7. Retired
    8. Unable to work (disabled)
14. Including you, how many people live in your household most of the time?____________
15. How many persons less than 18 years of age usually live in your home? _______________
16. What is your marital status?
    1. Now married
    2. Living with a partner / significant other
    3. Widowed
    4. Divorced
    5. Separated
    6. Never married
    7. Prefer not to answer
17. How much do you weigh in pounds? ________
18. How tall are you in feet and inches? ______ feet; ______ inches

**Section B. About your health (page 2-3):**

12. In general, how would you rate your overall health now?

- 1. Excellent
  2. Very good
  3. Good
  4. Fair
  5. Poor

1. Have you ever been told by a doctor or other health care professional that you have (choose all that apply)?
2. Type 1 or “juvenile diabetes” or “insulin dependent diabetes”
3. Type 2 or “adult diabetes”
4. Pre-diabetes or borderline diabetes
5. Gestational diabetes (or diabetes in pregnancy)
6. Don’t know/Not sure
7. Are you pregnant now?
8. Yes
9. No
10. Do not know
11. Prefer not to answer

**If YES to Q15 choice a or Q15 choice b, please ask 16, 17 and 18**

15. At what age were you first treated for diabetes? __________

16. Have you experienced any complications related to having diabetes? *Select all that apply.*

No complications

Complications with my eyes/vision (retinopathy)

Complications with my kidneys (nephropathy)

Complications with my feet (peripheral neuropathy)

Complications with my heart

Other complications: ________________________

17. Which prescribed diabetes medicines do you currently take?

*Select all that apply.*

No prescription medication

Prescribed pills

Insulin injections

Other types of injectable medications ________________________

18. Do you currently receive dialysis?

1. Yes
2. No

**Section C: Your Internet use (page 4-6)**

19. How did you hear about DiabetesSistersVoices? (select all that apply)

Friends or relatives

Twitters

Facebook

Newspaper, flyers or health magazines

Public health lectures

Others (please specify): ________________________

20. How often do you use email? Choose one.

1. Daily
2. Every few days
3. Weekly
4. Less than weekly
5. Never

22. Have you ever looked online for information about any health topics for you and your family, such as about a specific disease or treatment?

a. Yes🡪 22.1 Did you go online to look for information related to YOUR OWN health or medical situation or SOMEONE ELSE’S health or medical situation? *Check all that apply*.

1  My own

2  My child

3  Other family member

4  Friend

b. No

22.2 What websites do you use to look up health information?

Please specify: __________________________________

23. Have you gone online to find others who have health concerns similar to yours?

a. Yes

b. No

24. How confident are you in your ability to find helpful and useful health information on the internet?

a. Extremely confident

b. Somewhat confident

c. Neutral

d. Not very confident

e. Not confident at all

25. Do you use any social networking sites?

a. Yes 🡪 25.1 Please check all social networking sites that you have used:

b. Never 1  Facebook/MySpace

2  Twitter

3  Other______________________[write in]

25.2 How often do you use social networking sites?

a. Daily

b. Every few days

c. Weekly

d. Less than weekly

25.3 Thinking specifically about what you have done on social networking sites like Facebook and MySpace, have you ever used these sites to start or join a health related group?

a. Yes

b. No
