# Supplementary material for: DiabetesSistersVoices: Virtual Patient Community to Identify Research Priorities for Women Living With Diabetes
Source: J Med Internet Res. 2019 May 10;21(5):e13312. doi: 10.2196/13312 (PMC6533875; doi:10.2196/13312)
Supplement: Multimedia Appendix 5 [file jmir_v21i5e13312_app5.docx]

**Supplement E.** DiabetesSistersVoices End of Study Satisfaction Survey

Thank you for participating in the DiabetesSistersVoices research study.

1. Please check the option that best describes your experience:

|  | Strongly Disagree | Disagree | Neutral | Agree | Strongly Agree |
| --- | --- | --- | --- | --- | --- |
| I felt comfortable using the online process consent process to participate in the research study. | 0 | 1 | 2 | 3 | 4 |
| It was easy to participate in DiabetesSistersVoices online community. | 0 | 1 | 2 | 3 | 4 |
| Participation in DiabetesSistersVoices was confusing or frustrating. | 0 | 1 | 2 | 3 | 4 |
| I was comfortable expressing my views in the online discussion. | 0 | 1 | 2 | 3 | 4 |
| I felt like my opinions were heard by others in the community. | 0 | 1 | 2 | 3 | 4 |
| I felt I was supported by DiabetesSistersVoices community | 0 | 1 | 2 | 3 | 4 |
| The discussions gave me a better understanding of issues about women and diabetes. | 0 | 1 | 2 | 3 | 4 |
| I would recommend DiabetesSistersVoices to other women with diabetes or at risk for diabetes. | 0 | 1 | 2 | 3 | 4 |
| My participation in the community brought out questions I hadn't considered. | 0 | 1 | 2 | 3 | 4 |
| I was reluctant to share some of my views and experiences on the DiabetesSistersVoices. | 0 | 1 | 2 | 3 | 4 |

2. How satisfied are you overall with the following features of the DiabetesSistersVoices community?

|  | Very dissatisfied | Somewhat dissatisfied | Neither satisfied or dissatisfied | Somewhat satisfied | Very satisfied | I didn’t use this feature |
| --- | --- | --- | --- | --- | --- | --- |
| Consenting to participate in the study |  |  |  |  |  |  |
| Online survey about you and your health at the start of the study |  |  |  |  |  |  |
| Logging on to the website |  |  |  |  |  |  |
| Posting questions or comments |  |  |  |  |  |  |
| Searching for resources |  |  |  |  |  |  |
| Communicating with other members of the community |  |  |  |  |  |  |
| Topic of the week emails |  |  |  |  |  |  |

3. Please provide us with any additional comments you have about the DiabetesSistersVoices study:

Comment box

Thank you for your participation!
