# Supplementary material for: DiabetesSistersVoices: Virtual Patient Community to Identify Research Priorities for Women Living With Diabetes
Source: J Med Internet Res. 2019 May 10;21(5):e13312. doi: 10.2196/13312 (PMC6533875; doi:10.2196/13312)
Supplement: Multimedia Appendix 6 [file jmir_v21i5e13312_app6.docx]

| **Supplement G. Table.** Comparison of DiabetesSistersVoices study participants’ characteristics--those who only completed baseline social support and quality of life survey (and not end of study survey), N=205, compared with participants who completed both baseline and end of study social support and quality of life survey N=111. | | | |
| --- | --- | --- | --- |
|  | Completed only baseline survey  N=205 | Complete both baseline survey and end of study surveys  N=111 | *P*^a^ |
| Age - years^b^ | 49 (13.6) | 54 (12.6) | < .001 |
| Education^c^ |  |  |  |
| Less than high school or GED | 3 (1.5%) | 0 (0.0%) | .053 |
| HS or some college | 69 (33.7%) | 25 (22.9%) |  |
| College and above | 133 (64.9%) | 84 (77.1%) |  |
| Race^c^ |  |  |  |
| White | 117 (71.7%) | 98 (88.3%) | .88 |
| Black | 14 (6.8%) | 5 (4.5%) |  |
| Asian | 2 (1.0%) | 1 (9.0%) |  |
| Hispanic | 7 (3.4%) | 4 (3.6%) |  |
| Diabetes^c^ |  |  |  |
| Type 1 | 154 (75.1%) | 87 (78.4%) | .51 |
| Type 2 | 48 (23.4%) | 21 (18.9%) |  |
| Others | 3 (1.5%) | 3 (2.7%) |  |
| ^a^T-test for continuous variables and chi-square test for categorical variables.  ^b^All the continuous variables are displayed in mean (sd).  ^c^All the categorical variables are displayed in n (%). Percentage may not add up to 100% because of missing. | | | |
